# Supplementary material for: Patient-perceived factors influencing physical activity sensor use in stroke prevention and rehabilitation: A thematic synthesis protocol
Source: PLoS One. 2024 Apr 30;19(4):e0301983. doi: 10.1371/journal.pone.0301983 (PMC11060516; doi:10.1371/journal.pone.0301983)
Supplement: Attachment — Submitted filename: Response to Reviewers.docx [file pone.0301983.s002.docx]

27 December 2023

A/Prof Daswin De Silva

Academic Editor

PLOS ONE

Re: Response to Reviewers

Dear Prof. De Silva.

Please find below our responses to each of the points made in your decision letter, regarding our manuscript titled "Patient-perceived factors influencing physical activity sensor use in stroke prevention and rehabilitation: a thematic synthesis protocol".

Firstly, with respect to journal requirements (JR):

JR-1. The manuscript style was checked against the manuscript body formatting guidelines and the following amendments made: Level 2 headings in the Abstract were removed; two cited references were also removed from the Abstract; figure files were renamed according to the standard (i.e. nodes.eps to Fig1.eps; methodology.eps to Fig2.eps); “Discussion” heading was changed to “Results and discussion” with level 1 heading; “Supporting Information” was formatted level 1 heading and moved to after References as per the template; “Acknowledgements” was formatted with level 1 heading; “Conflicts of interest” was removed; “References” was formatted with level 1 heading and mixed case.
JR-2. The Data Availability statement has been amended to include an Open Science Framework link where datasets generated by the study will be made publicly accessible. This includes a Data Management Plan that describes data collection and analysis processes. The OSF project storage also includes a Supplementary Materials document that will be updated with search result sets and metrics, article screening and decision notes, quality and risk of bias assessments, final thematic node structure, and characteristics of included studies. Both documents will be made publicly available when data collection and analysis have concluded, and results have been published. We believe there are no ethical considerations that would restrict sharing these files.

Regarding Comments to the Author (CTA):

CTA-1. The manuscript has been amended to explain the rationale for the study, including research questions in the final paragraph of the Introduction.
CTA-2. The data collection and analysis procedures have been described more fully in the revised manuscript. Data collection and analysis are also described in the Data Management Plan.

Regarding Reviewer #1 comments (R1C):

R1C-1. The thematic synthesis process, well established in the qualitative research literature, will be recorded in reflexive notes generated by the two reviewers during this highly collaborative phase of the study. The summaries and preliminary analysis will be created in Microsoft Excel, based on coding density counts exported from NVivo. Summary measures will identify major and minor themes and these metrics will also be used to create sunburst charts in Excel for barrier and facilitator themes. The manuscript has been amended accordingly.
R1C-2. Regarding the recommendation to measure inter-rater reliability during screening, as there will be only two reviewers, we propose instead to calculate a simple agreement rate, and have updated the protocol to include this. Additionally, a pilot test will be performed, screening example article(s) against the inclusion and exclusion criteria to ensure mutual understanding between reviewers. Discrepancies in screening and coding will be resolved through discussion, or the engagement of a third party where consensus cannot be reached. Standard data extraction and screening forms will be used (Supplementary Materials, sections C & E). Detailed records of the screening process will be maintained, as described in the Supplementary Materials document.
R1C-3. The a priori coding framework (node structure) is illustrated in Fig 1 and described in the manuscript and Supplementary Materials.

Regarding Reviewer #2 comments (R2C):

R2C-1. Included citations for stroke risk factors, and PA guidelines.
R2C-2. Updated to clarify that efficacy refers to stroke prevention and rehabilitation.
R2C-3. Updated to clarify that context is with respect to the efficacy of PA sensor-based interventions.
R2C-4. The bibliographic search was expanded to 31 December 2023, and the projected results availability shifted to June 2024.
R2C-5. Rationale for age range explained with citations in the second paragraph of the introduction.
R2C-6. “Analytical richness” defined, with citation.
R2C-7. “Axial coding” defined, with citation.
R2C-8. Research questions have been added to the last paragraph of the Introduction.

Thank you for your comments and considering our manuscript.

Sincerely,

Paul Harris

University of Melbourne

Parkville, Victoria, 3010, Australia
